# Ferroptosis-related local immune cytolytic activity in tumor microenvironment of basal cell and squamous cell carcinoma

**DOI:** 10.18632/aging.204057

**Published:** 2022-05-02

**Authors:** Jianqiao Wang, Dong Xie, Hongxuan Wu, Yuchen Li, Chuan Wan

**Affiliations:** 1 Department of Dermatology, The First Affiliated Hospital of Nanchang University, Nanchang 330006, China

**Keywords:** basal cell carcinoma, squamous cell carcinoma, local immune cytolytic activity, tumor microenvironment, ferroptosis

## Abstract

Background: Ferroptosis, a recently discovered form of cell death, whose role in basal cell carcinoma (BCC) and squamous cell carcinoma (SCC) has not been well disclosed. To improve our understanding of the differences in tumor progression and therapeutic effects between BCC and SCC, and to find potential therapeutic targets, this study systematically analyzed ferroptosis-related genes (FRGs) and their associated local immune cytolytic activity (LICA) and tumor microenvironment (TME) metabolic function differences.

Methods: Two bulk RNA-seq datasets, GSE7553 and GSE125285, from the Gene Expression Omnibus database were compared within and between groups to screen for common differentially expressed genes (DEGs) for enrichment analysis. The currently recognized FRGs in DEGs gene set were selected as the targets to analyze their correlation and difference in LICA and TME metabolic functions. And validated using immune cell populations from another single-cell RNA-seq (scRNA-seq) dataset (GSE123813) to accurately understand the difference in LICA. All of the gene sets for functional enrichment analysis comes from published results and MSigDB database.

Results: Ten FRGs were used to further analyze the differences in LICA and TME metabolic functions between BCC and SCC. In the SCC samples, LICA (e.g. Treg, CCR, Cytolytic activity, etc.) and TME metabolic functions (e.g. lipid and energy, etc.) were significantly related to ferroptosis genes (e.g. SLC1A5, CD44, NQO1, HMOX1 and STEAP3), and the ferroptosis potential index were also significantly higher than that in the BCC samples. Finally, based on these ten FRGs and related enrichment results, we postulated a model of NQO1 homeostasis regulated by FRGs during induction of ferroptosis in SCC.

Conclusions: The results showed that three FRGs, SLC1A5, CD44 and NQO1, have significant potential in targeted therapies for SCC chemotherapy resistance. And two FRGs, STEAP3 and HMOX1, formed a synergistic effect on the occurrence of ferroptosis in tumor cells. Our findings can be used as the main research materials for metastasis and chemotherapy resistance in SCC patients.

## INTRODUCTION

Research on tumor cell death has been the main goal of cancer treatment, and the mechanism of acquired or intrinsic resistance to apoptosis and necrosis of cancer cells has become a key research object in the field of tumor treatment. After more than 30 years of exploration of the mechanism of programmed death of cancer cells, necrotic apoptosis has been confirmed to be a typical form of cancer cell necrosis, which is mediated by receptor interacting protein kinase 3 (RIPK3) and mixed-lineage kinase domain-like synergism [1, 2]. Ferroptosis, iron-catalyzed necrosis, is a recently discovered form of programmed cell death [3], in which cell necrosis is regulated by over-peroxidation of polyunsaturated fatty acids (PUFAs). Increased iron dependence is responsible for cancer cells not being able to undergo typical necrotizing apoptosis [4, 5] and ultimately fail to initiate the death program in the normal human metabolic environment, which is an important reason for cancer cells proliferation and evasion of immune attack or drug treatment.

Ferroptosis-related genes (FRGs) in a variety of tumors (such as hepatocellular carcinoma [6], kidney renal clear cell carcinoma [7], lung adenocarcinoma and esophageal carcinoma, etc. [8]) significantly affect the survival time of tumor patients, and are related to a variety of immune cells and immune-related signaling pathways [9, 10]. However, the association of FRGs with local immune cytolytic activity (LICA) and tumor micro-environmental (TME) metabolism in basal cell carcinoma (BCC) and squamous cell carcinoma (SCC) has not been reported. In this study, we compared the expression differences of FRGs in the LICA and related immune pathways and metabolism function between BCC and SCC, and found some important immune signaling pathways in SCC tumors are significantly up-regulated along with high-level cytolytic activity of immune cells.

## MATERIALS AND METHODS

### Sources of original data

The datasets used to explore the genes expression characteristics of BCC and SCC were retrieved from two gene expression matrices GSE7553 [11] and GSE125285 [12] in the Gene Expression Omnibus (GEO) database. Genes expression matrix data from 15 BCC, 11 SCC and 4 normal samples in the GSE7533 dataset and 25 BCC, 10 SCC and 35 adjacent normal tissue samples in the GSE125285 dataset were used in this study. To verify and accurately understand the difference in the role of LICA in BCC and SCC tissues, this study extracted the immune T cell communities from a single-cell RNA-seq (scRNA-seq) dataset GSE123813 [13] for further validation.

### Functional enrichment analysis of DEGs gene set

To reduce the impact of sample batch effect between different studies, the normalized function of limma (version 3.46) program was used to normalize the data matrices in the two studies respectively. And the differentially expressed genes (DEGs with P.adj < 0.05, log2FoldChange > 1) in BCC and SCC samples were selected as the DEGs gene set. Genes function enrichment analysis was performed using gene set variation analysis (GSVA, version 1.42), including differences in correlation between LICA or TME metabolic function and FRGs. In addition, the DEGs gene set was used to compare between the two studies with the gene expression matrix of normal or adjacent normal tissues and the genes enrichment analysis results of BCC and SCC.

### Sources of ferroptosis genes, LICA and TME gene set

The LICA related gene set used for single sample gene set enrichment analysis (ssGSEA) comes from the attached files of the cytolytic activity of 18 TCGA tumors by Rooney et al. [14], including 15 immune cells and 14 immune cell-related functions used to describe the correlation difference of FRGs. Differences in TME changes related to FRGs refers to Peng et al. [15] based on the 7 metabolic expression subtypes of 33 TCGA tumor classifications: including amino acid, TCA cycle, nucleotide, energy, carbohydrate, lipid, and vitamin and cofactor metabolism. The 63 FRGs were collected from the review of Hassannia et al. [4] and the results of related tumor studies [16]. Tumor associated epithelial–mesenchymal transition (EMT) activity and reactive oxygen species (ROS) function gene sets were retrieved from MSigDB database [17].

### Methods and instruments for statistical analysis

The overall score share (OSS) of all samples is a score calculated based on sample size and gene expression levels in selected gene sets to assess the activity of biological processes in different sample types. OSS was calculated by formula $\text{OSS}=\text{m}/\text{n}*\sum_{\text{i}}^{\text{n}}{\text{e}}^{{\text{S}}_{\text{i}}}/\sum_{\text{j}}^{\text{m}}\sum_{\text{i}}^{\text{n}}{\text{e}}^{{\text{S}}_{\text{i}}}$ (m is the number of groups, n is the number of samples in each group, S is the GSVA score of a single sample, j=i=1). Finally, combined with the results of the Pearson correlation test, this study established a correlation-connected OSS (ccOSS) network model diagram to better understand the correlation and difference in the gene set enrichment functions of different tissue types.

The ferroptosis potential index (FPI), proposed by Liu et al. [8] to explore the role of ferroptosis-related factors or biological processes, is a model based on the difference between positive and negative scores of ssGSEA enrichment results. In this study, the Student’s t-test or Wilcox-test was used for the comparison between the two groups of samples, and ANOVA was used for the comparison in the three groups of samples, and P <0.05 was considered statistically significant. Seurat 4.0 [18] and GSVA 1.42 [19] were used in multimodal single-cell and gene set enrichment analysis respectively. All analytical processes were performed in R-4.0.4 (R Foundation for Statistical Computing, Vienna, Austria) environment.

## RESULTS

### Ten ferroptosis genes included in the DEGs gene set

**Figure 1:**
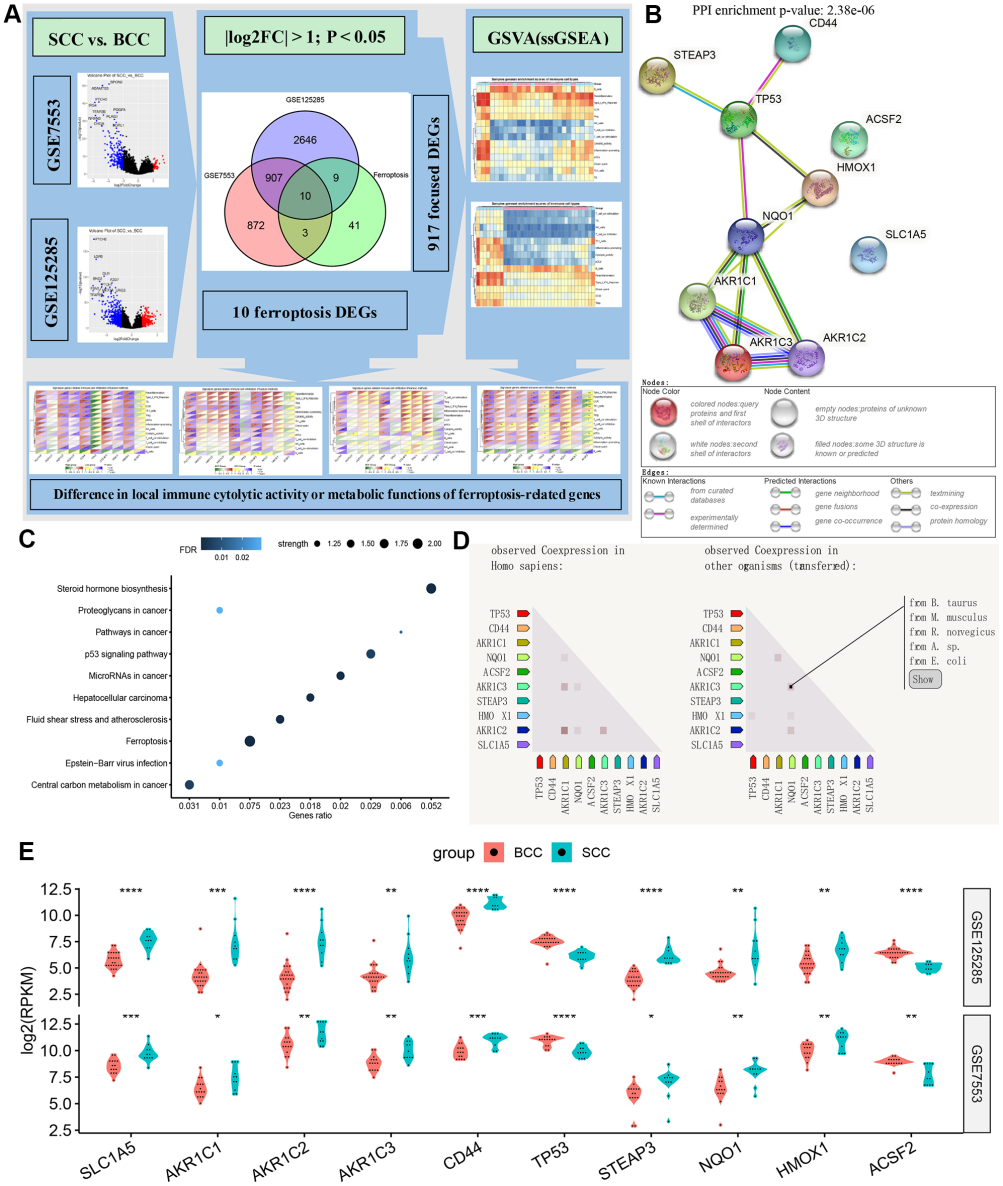
**Overview of ferroptosis genes.** (**A**) Flow chart of DEG gene set and iron death gene acquisition. (**B**–**D**) The protein-protein interaction network diagram, biological functions and (homologous) co-expression results of ferroptosis genes. (**E**) Differences in the expression of ferroptosis-related genes between BCC and SCC in the two data sets. (*P<0.05,**P<0.01, ***P<0.001, ****P<0.0001).

As shown in Figure 1A, in GSE7553 and GSE125285, BCC vs. SCC obtains 1792 and 3572 DEGs, respectively. In the total gene set consisting of 917 DEGs (P.adj < 0.05, log2FoldChange > 1), there are 10 FRGs, including SLC1A5, AKR1C1, AKR1C2, AKR1C3, CD44, TP53, STEAP3, NQO1, HMOX1 and ACSF2. Notably, members C1, C2, and C3 of Aldo-Keto reductase family 1 (AKR1) appear in the DEGs gene set. The results of the protein-protein interaction (PPI) network related to these 10 genes are shown in Figure 1B. Proteins AKR1 and NAD(P)H quinone dehydrogenase 1 (NQO1) are directly connected with a enrichment P value is 2.38e-6. These probably indicate that the ferroptosis gene-related redox reactions is involved in tumor cell microenvironment metabolism and plays an important role in BCC and SCC. Further genes function enrichment analysis showed (Figure 1C) that ferroptosis, central carbon metabolism in cancer and steroid hormone synthesis have higher strength (this measure describes how large the enrichment effect is.), followed by tumor-related *p53* and microRNA alteration. Based on the RNA expression pattern and the co-expression score of protein co-regulation, Figure 1D depicts the co-expression of these 10 FRGs in other species (such as B. taurus, M. musculus, etc.). Apart from the genes TP53 and ACSF2, the relative expression of other FRGs were significantly higher in the SCC group than in the BCC group (Figure 1E).

### Functional enrichment differences of DEGs gene set

In the gene expression DEGs gene set of BCC and SCC, some typical biological signal pathways showed obvious differences (Supplementary Figure 1A). For example, the OSS of basal cell carcinoma, melanogenesis, endocytosis and WNT signaling pathway were higher in the BCC group than in the SCC group. On the contrary, the OSS of metabolic pathways and tightly junction were higher in the SCC group than in the BCC group. In addition, the GSEA results of DEGs gene set ranking score (fitted log2 (RPKM)) (Supplementary Figure 1B) also showed that the NES and P values of typical biological signaling pathways of BCC and SCC were significantly different. For example, in GSE7553 dataset, pathway “basal cell carcinoma” was significant only in the BCC group (P < 0.05), while “metabolic pathways” was significant only in the SCC group (P < 0.05). And in GSE125285 dataset, pathways “WNT signaling pathway”, “melanogenesis” and “basal cell carcinoma” were significant only in BCC group (P<0.05), while “endocytosis” was significant only in SCC group (P<0.05). To better describe the difference in the role of DEGs in the key biological signals of BCC and SCC, important KEGG pathway diagrams annotated by the gene expression level of the DEGs gene set were supplemented in attached file (Supplementary File 1).

### Correlation of FRGs and LICA

**Figure 2:**
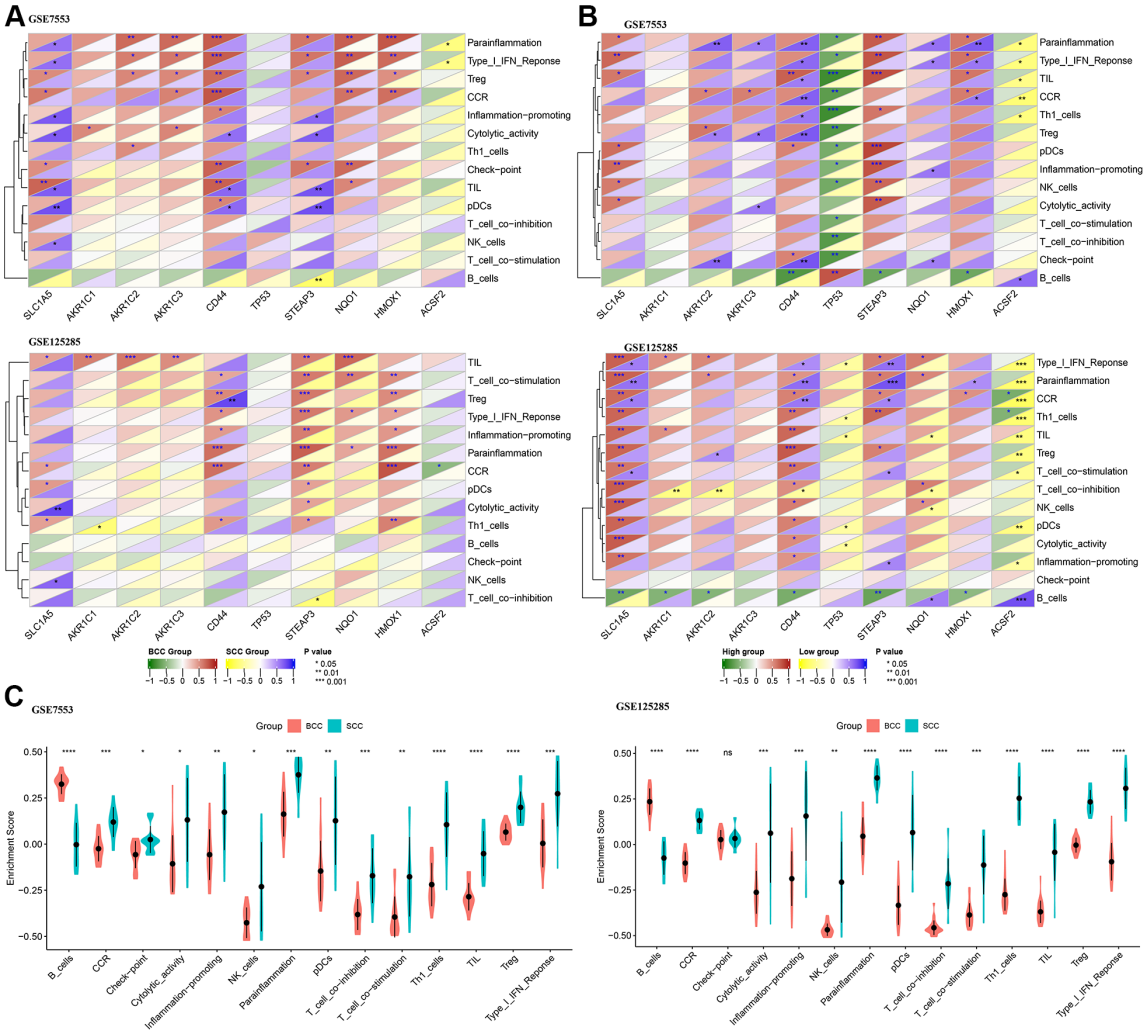
**Correlation difference between ferroptosis genes and local immune cytolytic activity (LICA).** (**A**) Difference in Pearson correlation of LICA and ferroptosis genes between BCC and SCC. (**B**) Difference in Pearson correlation of LICA and ferroptosis genes higher/lower expression level. (**C**) Comparison of LICA results between BCC and SCC. (*P<0.05,**P<0.01, ***P<0.001, ****P<0.0001).

As shown in Figure 2A, CD44, STEAP3, NQO1 and HMOX1 have significant positive correlation with immune cells (Treg) or function (CCR, Parainflammation and Type I IFN Response) in the BCC group. The gene SLC1A5 has a significant positive correlation with immune cells (NK cells) or function (Cytolytic activity) in the SCC group. However, the correlation results of other FRGs with immune cells or function are inconsistent in the two datasets. To further understand the relationship between FRGs expression level and LICA, based on the gene expression level in each FRG of the tumor tissue (BCC and SCC), the correlation results between each gene’s median expression level and the high versus low expression groups are shown in Figure 2B, which illustrated correlation results between these 10 FRGs and LICA from another perspective.

Furthermore, by comparing the LICA enrichment results of BCC and SCC tissue samples (Figure 2C), only the local immune activity of B cells was significantly reduced in the SCC group, while other immune cells or function scores were significantly higher than those of the BCC group, such as cytolytic activity, tumor infiltrating lymphocytes (TIL), etc.

### Correlation of FRGs and metabolic function of TME

Subsequently, the TME metabolic function differences between the BCC and SCC groups, and between the high versus low expression groups of FRGs were compared. As shown in Supplementary Figure 2A, lipid metabolism in the BCC group was significantly positively correlated with genes AKR1C2, AKR1C3, NQO1 and HMOX1 in both datasets. Such a relationship was also found in Supplementary Figure 2B, which was grouped by median of gene expression level. However, other ferroptosis gene-related metabolic functions presented inconsistent results between the two datasets. And the enrichment score of TME metabolic functions (except carbohydrate and TCA cycle) in the SCC group was significantly higher than that in the BCC group (Supplementary Figure 2C).

### Difference in ccOSS

**Figure 3:**
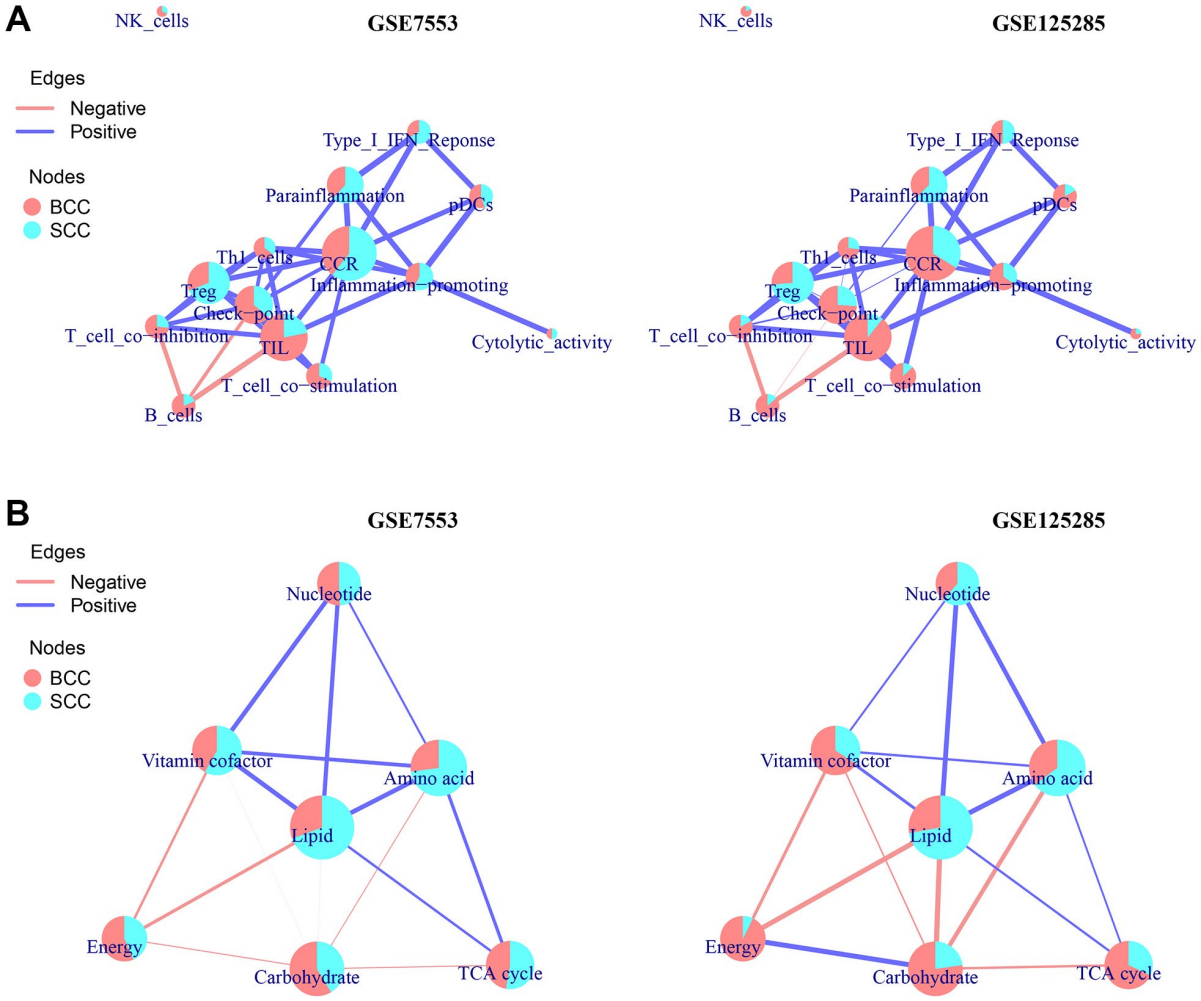
**A network model of correlation-connected overall score share (ccOSS) of LICA and TME between BCC and SCC.** (**A**, **B**) The ccOSS network interaction of BCC and SCC in LICA and TME enrichment results, respectively. (The connection of the node is determined by the shared genes in the node function, the size of the node is determined by the number of genes in the enriched gene set, and the overall score share (OSS) in node is calculated according to the formula $\text{OSS}=\text{m}/\text{n}*\sum_{\text{i}}^{\text{n}}{\text{e}}^{{\text{S}}_{\text{i}}}/\sum_{\text{j}}^{\text{m}}\sum_{\text{i}}^{\text{n}}{\text{e}}^{{\text{S}}_{\text{i}}}$, with m represent the number of groups, n represent the number of samples in each group, S is the GSVA score of the sample, and j=i=1. The thickness of the connection line is determined by the correlation coefficient between the nodes, and there are positive and negative differences).

Finally, a ccOSS network model was constructed to compare the shared scores of each enrichment result, including the immune cells or function of LICA and the metabolic function of TME, in the overall sample set of BCC and SCC. As shown in Figure 3A, the network structure of most LICA results and the occupancy ratio of each node in the two datasets were almost the same, except for CCR, inflammation promoting, cytolytic activity and type I IFN response. Similar to the results obtained above, in the ccOSS network model diagram, we found that Treg and Parainflammation had higher occupancy rates in the SCC group, while NK cells, Th1 cell, T cell co- inhibition/stimulation, B cells and TIL had higher occupancy rates in BCC group.

Similarly, in the TME functional network diagram (Figure 3B), in addition to vitamin cofactor and TCA cycle, nucleotide, amino acid and lipid metabolism had higher shared scores in the SCC group, while energy and carbohydrate metabolism had higher shared scores in the BCC group. Notably, we found that the enrichment results of KEGG, LICA and TME in adjacent normal tissues were significantly different from those in normal tissues (Supplementary Figure 3), and their shared scores in the ccOSS network diagram even exceeded that of BCC or SCC samples, which is why this study did not directly compare adjacent/normal tissues in the two datasets.

### Difference of LICA in immune cell subsets

**Figure 4:**
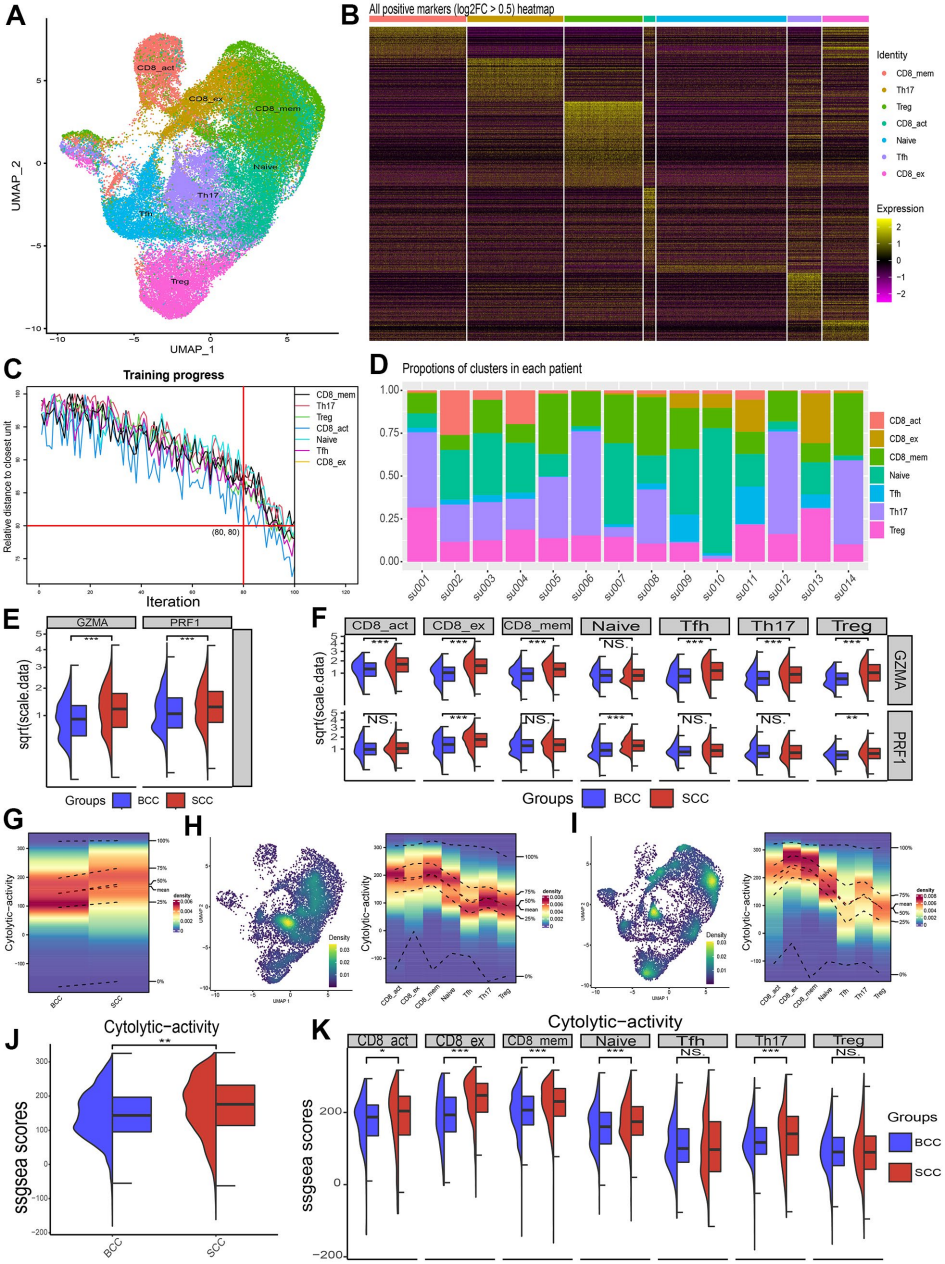
**Differences in cytolytic activity of subpopulations of immune cells in single-cell samples from BCC and SCC patients.** (**A**) Umap of T cell subpopulations’ distribution. (**B**) Heat map of relative expression of T cell subgroup-specific DEGs (p < 0.05, log2FC > 0.5). (**C**) Relative distance test of the training progress with 100 iterations of each cluster cells. (**D**) The proportion of T-cell subsets in each patient sample. (**E**, **F**) Differences in relative expression of the genes GZMA and PRF1 in BCC, SCC and T cell subpopulations. (**G**–**I**) Density profiles of cytolytic activity in BCC (**H**), SCC (**I**) and individual T cell subsets. (**J**, **K**) Wilcox test for differences in cytolytic activity of T cells and its subpopulations in BCC and SCC samples.

All of the above, LICA showed significant enrichment differences between BCC and SCC tissue samples. However, the disadvantage was that it is difficult to accurately described the important role of the cytolytic activity of immune cells on the TME due to the mixed cell types in tissue samples. Therefore, this study extracted the T cell populations (include 28554 cells) of BCC and SCC from a public scRNA-seq dataset (GSE123813). Then we classified T cell into 7 subsets according to the annotation results of the data provider (Figure 4A), including CD8 activation, CD8 exhaustion, CD8 memory, Naive, Tfh, Th17 and Treg. Subset-specific marker genes (Figure 4B) and a relative distance test of training progress at 100 iterations per cluster unit (Figure 4C) indicated that the T cell classificatory results were reliable and stable. Figure 4D lists the proportional distribution characteristics of T cell subsets in single cell samples of BCC and SCC.

Afterwards, two key cytolytic effector genes, GZMA and RPF1, were found significantly higher in SCC than in BCC in T cell community (Figure 4E), and also in most T cell subsets (Figure 4F). Figures 4G–4I illustrated the density distribution (ssGSEA method) results of the cytolytic activity of BCC (H), SCC (I) and T cell subsets, respectively. Figures 4J, 4K illustrated the significant differences in T cell subsets between BCC and SCC through Wilcox test.

## DISCUSSION

To our knowledge, this study systematically investigated the differences in ferroptosis gene-related LICA and metabolic functions of TME in BCC and SCC for the first time. As listed above, this report not only obtained LICA (immune cell or function) and TME metabolic functions that were closely related to FRGs, and confirmed that the LICA of immune T cell in SCC was significantly higher than that in BCC.

The comparative results of ccOSS in this study showed that the metabolism of nucleotides, lipids and amino acids were more important in the SCC microenvironment. Among the 10 FRGs analyzed, the genes SLC1A5(alias ASCT2) and ACSF2 controlled amino acid transport [20] and fatty acid metabolism [21], respectively. Current studies on SLC1A5 in tumor cells have shown that increased SLC1A5 expression promotes glutamine dependence and antioxidant stress, affects the components of immune cell infiltration, and is associated with poor tumor prognosis [22–24]. Preclinical models have shown that pharmacological blockade of SLC1A5-dependent glutamine transport can exert anti-tumor effects [25]. Based on the existing evidence, SLC1A5 is an important target in the process of tumor cell progression, and new evidence shows that SLC1A5 inhibitors can provide considerable therapeutic effect for cancer treatment [26]. ACSF2 is a member of the Acyl-CoA synthetase family, mainly distributed in mitochondria and cytoplasm. It catalyzes the initial reaction in fatty acid metabolism by forming thioesters with CoA, and plays a role in adipocyte differentiation [21, 27, 28].

**Figure 5:**
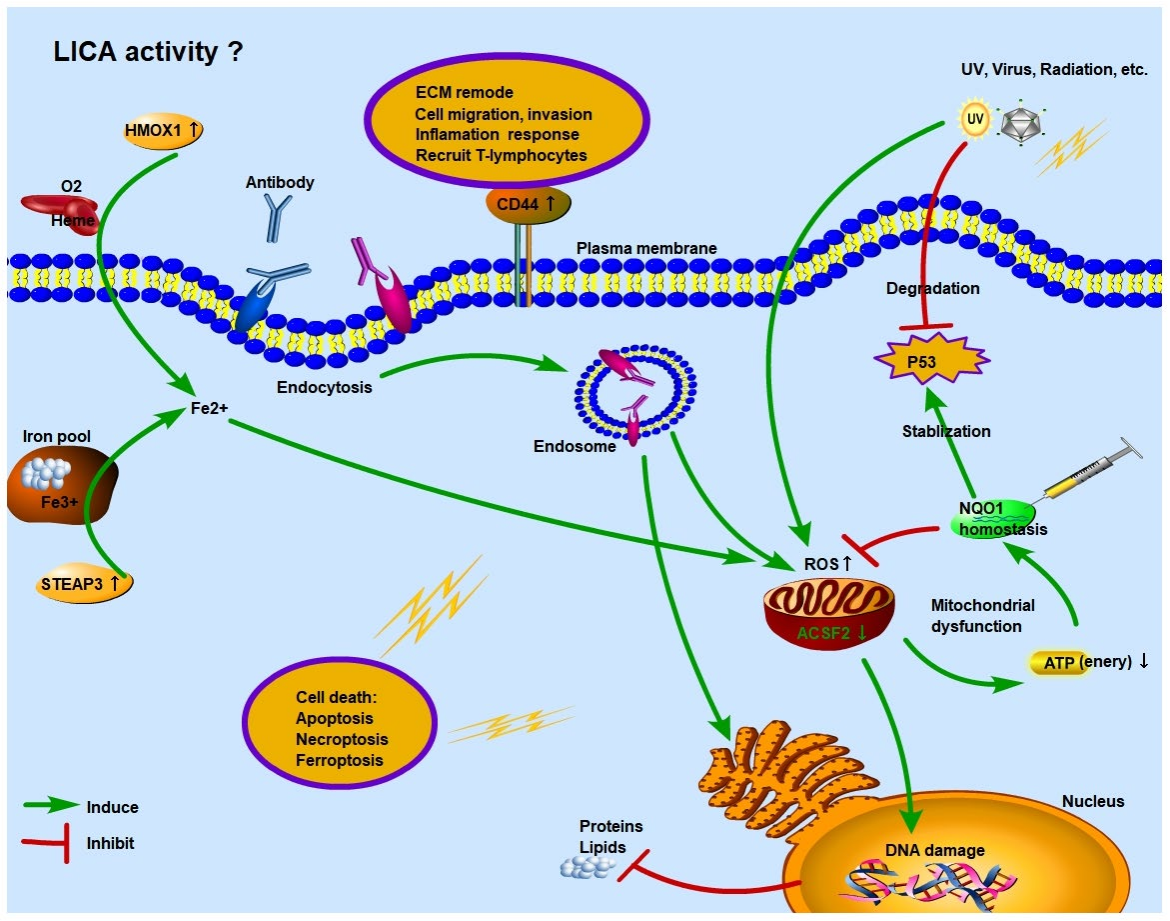
**Schematic diagram of function regulation mode of ferroptosis genes in SCC.** Under the stress of UV, virus or radioactive factors, normal cells lose the control of tumor suppressor gene TP53 and proliferate malignantly. At the same time, excessive activation of ROS function in the intracellular environment causes stress damage to mitochondria and endoplasmic reticulum. The imbalance of NQO1 homeostasis in SCC tumor cells cannot guarantee the stabilization of P53 and the inhibitory effect of ROS. Moreover, under immune surveillance, the cytolytic activity of local immune cells is enhanced, and endocytosis is formed through antigen-antibody binding, which further induces cancer cells undergo apoptosis or necrosis. However, in the early stage, under the regulation of NQO1 homeostasis, some cancer cells adapt to the higher ROS environment. These “super tumor cells” accelerate the progression of SCC and increase the degree of malignancy. Ferroptosis is a process of cell necrosis catalyzed by iron. When a large amount of Fe2+ accumulates in the intracellular environment, it will synergistically increase the function of ROS. The overactivation of HMOX1 and STEAP3 increase Fe2+ levels through Heme and iron pool, respectively.

In addition, we noticed that energy metabolism was weakened in SCC. Therefore, we hypothesized a model of NQO1 homeostasis that regulates ROS function in the intracellular environment (Figure 5). NQO1 plays a key role in anti-oxidation (inhibiting ROS function) and stabilization of p53 [29, 30]. Loss of NQO1 regulation could induce p53 degradation and relax the restraint on ROS function. High levels of ROS may promote the adaptive survival of cancer cells and protect cancer cells from chemotherapeutic or cytotoxic treatments. These “super tumor cells” accelerate the progression of SCC and increase the malignancy of tumor. Currently, NQO1-mediated chemotherapy resistance has been confirmed in cholangiocarcinoma tumor cells [31], and the targeted metabolism drug KP372-1 mediated by NQO1 has effectively inhibited tumor growth in xenograft models [32].

As we found above, two FRGs, HMOX1 and STEAP3, were significantly upregulated in SCC tissues, both of which could promote Fe3+ to Fe2+ transformation [33, 34] (Figure 5). And FPI was significantly higher in SCC tissues than in BCC and adjacent/normal tissues, as a result, we compared the relationship between ferroptosis and BCC or SCC. Excessive expression of HMOX1 can catalyze the degradation of heme to Fe2+, biliverdin and carbon monoxide, and ferroptosis sensitivity also changes during the process of lipid peroxidation [35]. Excessive expression of STEAP3 is the culprit in releasing Fe3+ from the intracellular iron pool [36], and its metal reduction function further increases the concentration of Fe2+ in intracellular environment. Therefore, under the synergistic effect of HMOX1 and STEAP3, the increased concentration of Fe2+ in intracellular environment causes the imbalance of iron homeostasis, and its participation in free radicals and lipid peroxidation promotes the occurrence of cell ferroptosis.

Tumor cell metabolic dysfunction also alters the immune and inflammatory responses. CD44, an upregulated FRG in SCC, is highly correlated with immune function in this study, and plays an important role in biological processes such as inflammation response, extracellular matrix remodeling, and tumor cell invasion and metastasis, and EMT [37–40]. At present, tumor suppression therapy targeting CD44 can inhibit tumor angiogenesis and cell metastasis [41, 42], and protect cells from ROS damage by activating downstream MAPK signals [43]. Over-expressed CD44 has a positive regulatory relationship with LICA immune cells and functions (Treg, TIL, Th1 cells, etc.) in SCC tissues. Therefore, CD44 is of great significance as a targeted therapy for SCC metastasis and drug resistance.

Significantly different from SCC, the results of LICA, TME and ROS function enrichment in BCC tissues were very close to those in adjacent/normal tissues (Supplementary Figure 4A, 4B, 4D), and the expression levels of 8 genes out of the 10 FRGs were lower than those in adjacent/normal tissues (Supplementary Figure 4C). This outcome prompted us to further investigate the reasons behind it, but the results revealed some shortcomings in this study that should not be ignored. In both of the two GSE dataset and related literature, the time and status of the patient’s BCC and SCC tissue acquisition (e.g., whether chemotherapy or targeted therapy was received) are not provided, which was critical to our discussion of the hypothetical NQO1 homeostasis model. Moreover, in both datasets, the expression of NQO1 in SCC did not significantly increased or decreased compared with that in adjacent/normal tissues (Supplementary Figure 4C), but it was significantly decreased in BCC than in normal tissues (not adjacent normal tissues). Unfortunately, when we contacted the authors of these datasets, we got no response.

In conclusion, under the protective effects of cellular immune surveillance, NQO1 homeostasis and iron homeostasis, most damaged cells undergo natural apoptosis or necrosis, but those “super tumor cells” must be cleared in time through reasonable treatments to avoid deterioration and metastasis. Therefore, the potential target FRGs, SLC1A5, CD44 and NQO1, for tumor resistance treatment have important research value in the treatment of SCC drug resistance. *In vitro* and preclinical trials should be carried out in the future to further evaluate the therapeutic effect of these gene targets.

### Date availability statement

The data that support the findings of this study are available from the Gene Expression Omnibus database. Bulk RNA-seq datasets (GSE7553 and GSE125285) and single-cell RNA dataset (GSE123813). Complete analysis code was deposited in the platform of gitee (https://gitee.com/plainTHS/bscc).

## Supplementary Material

Supplementary Figures

Supplementary File 1
