# Supplementary material for: Ferroptosis-related local immune cytolytic activity in tumor microenvironment of basal cell and squamous cell carcinoma
Source: Aging (Albany NY). 2022 May 2;14(9):3956–72. doi: 10.18632/aging.204057 (PMC9134950; doi:10.18632/aging.204057)
Supplement: Supplementary Figures [file aging-14-204057-s001.pdf]

SUPPLEMENTARY FIGURES

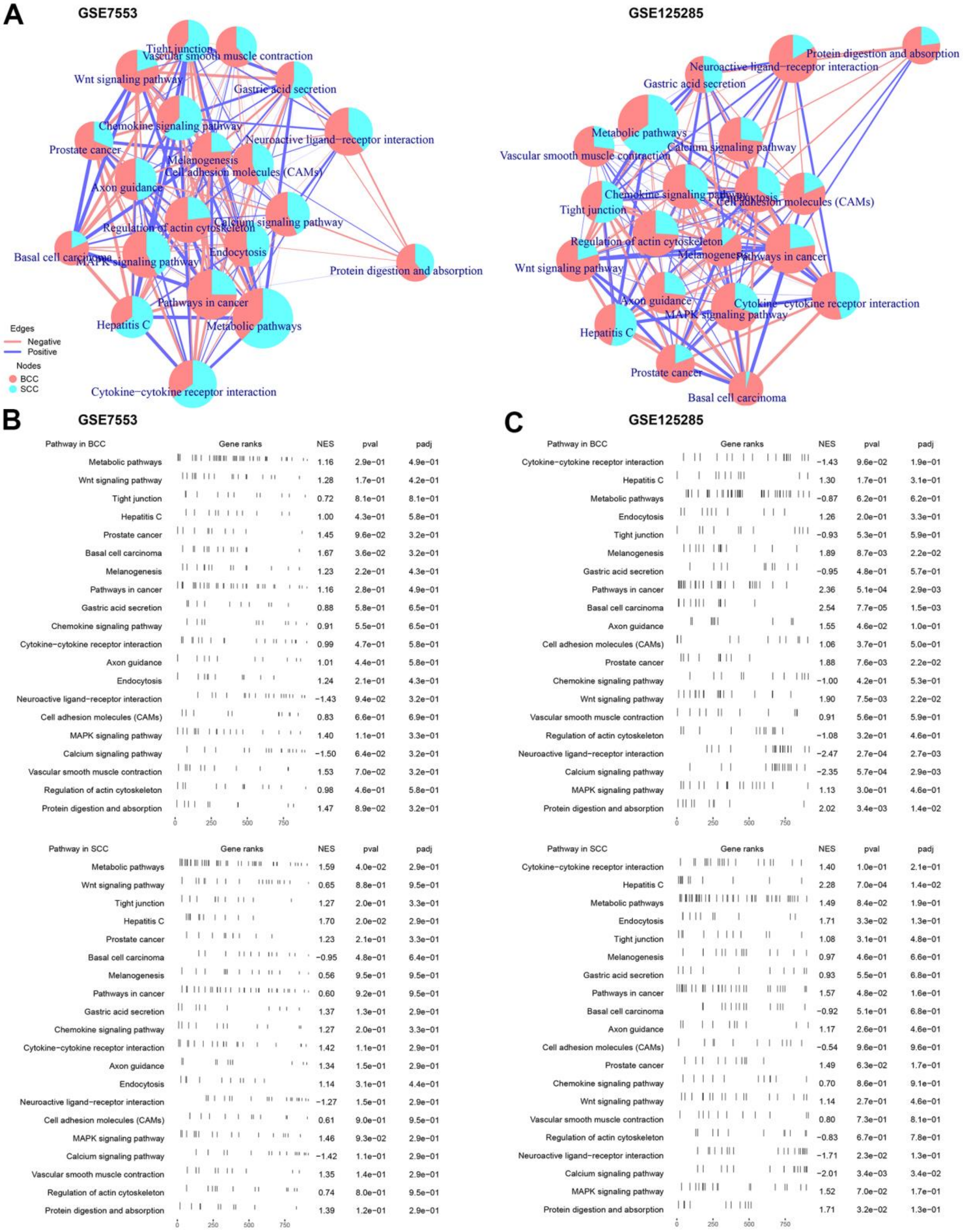

Supplementary Figure 1. KEGG and GSEA enrichment results of DEGs geneset. (**A**) The top 20 KEGG pathways interactive network for the overall score share of BCC and SCC. (**B**, **C**) GSEA enrichment results of BCC and SCC in GSE7533 and GSE125285.

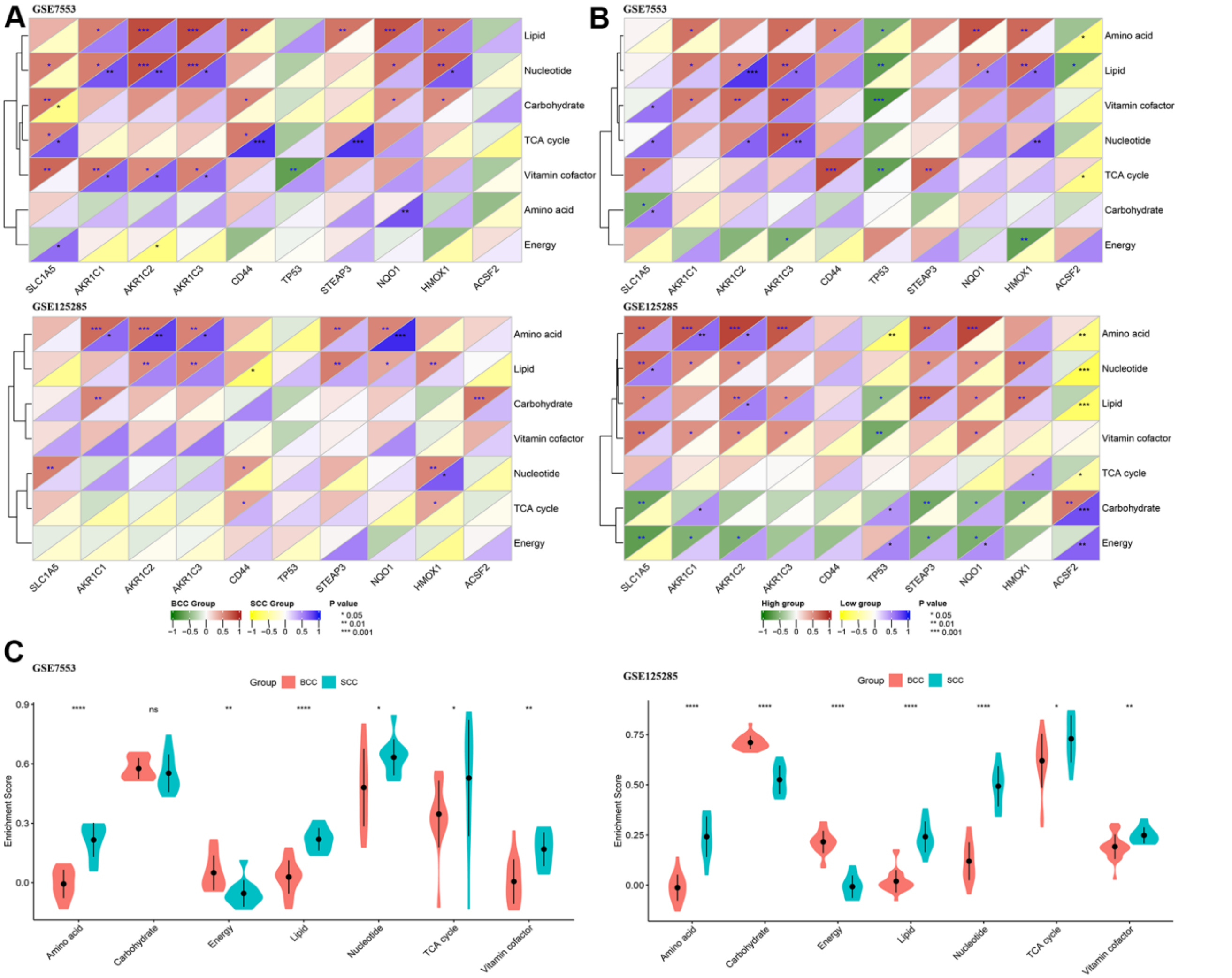

Supplementary Figure 2. Correlation difference between ferroptosis genes and tumor microenvironment (TME) metabolic functions. (**A**) Difference in Pearson correlation of TME metabolic functions and ferroptosis genes expression between BCC and SCC. (**B**) Difference in Pearson correlation of ferroptosis genes higher/lower expression level and TME. (**C**) Comparison of TME metabolic function between BCC and SCC. (*P<0.05,**P<0.01, ***P<0.001, ****P<0.0001).

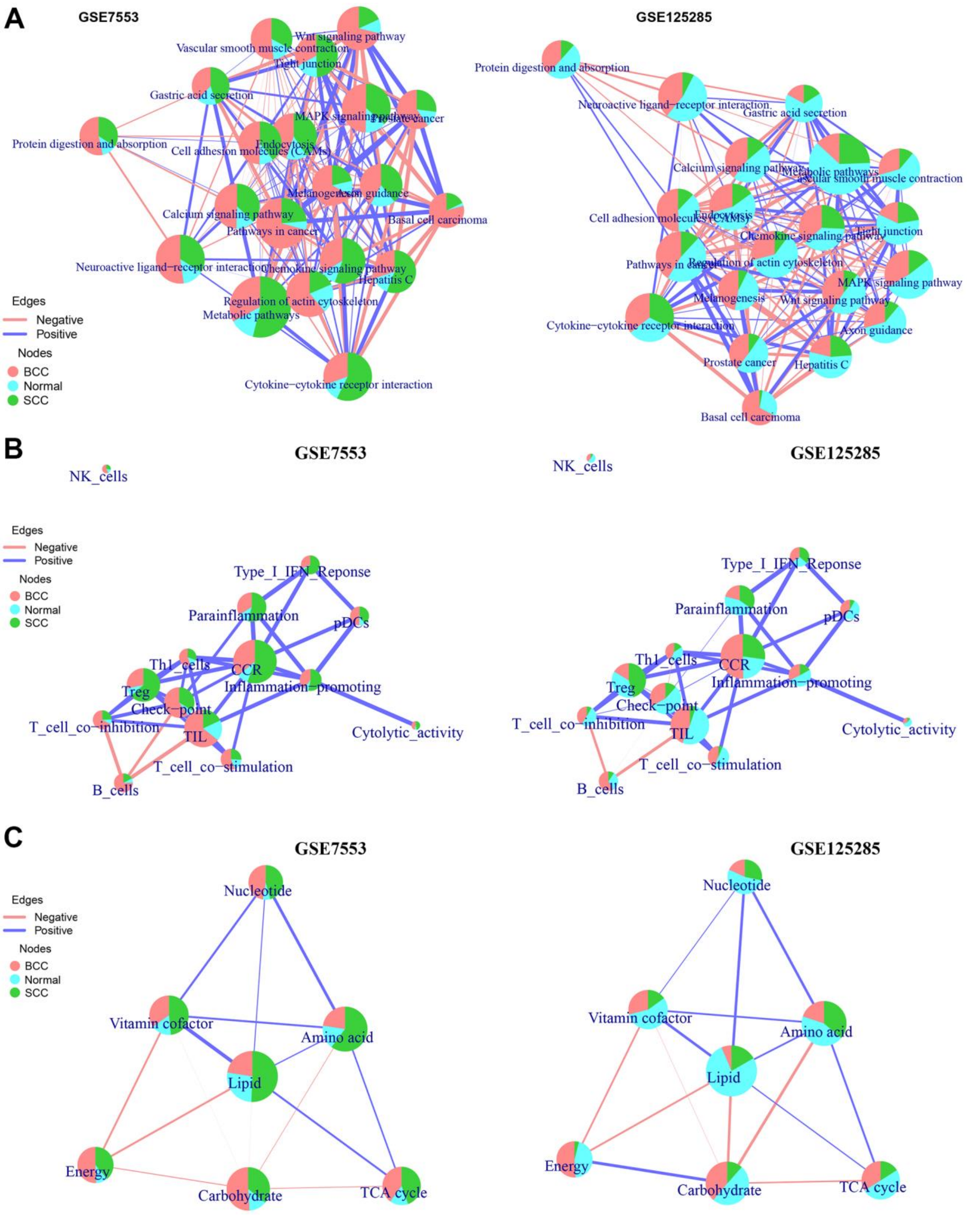

Supplementary Figure 3. Enrichment results of KEGG (**A**), LICA (**B**) and TME metabolic function (**C**) in overall score share of interactive network of BCC, SCC and (adjacent) normal tissues.

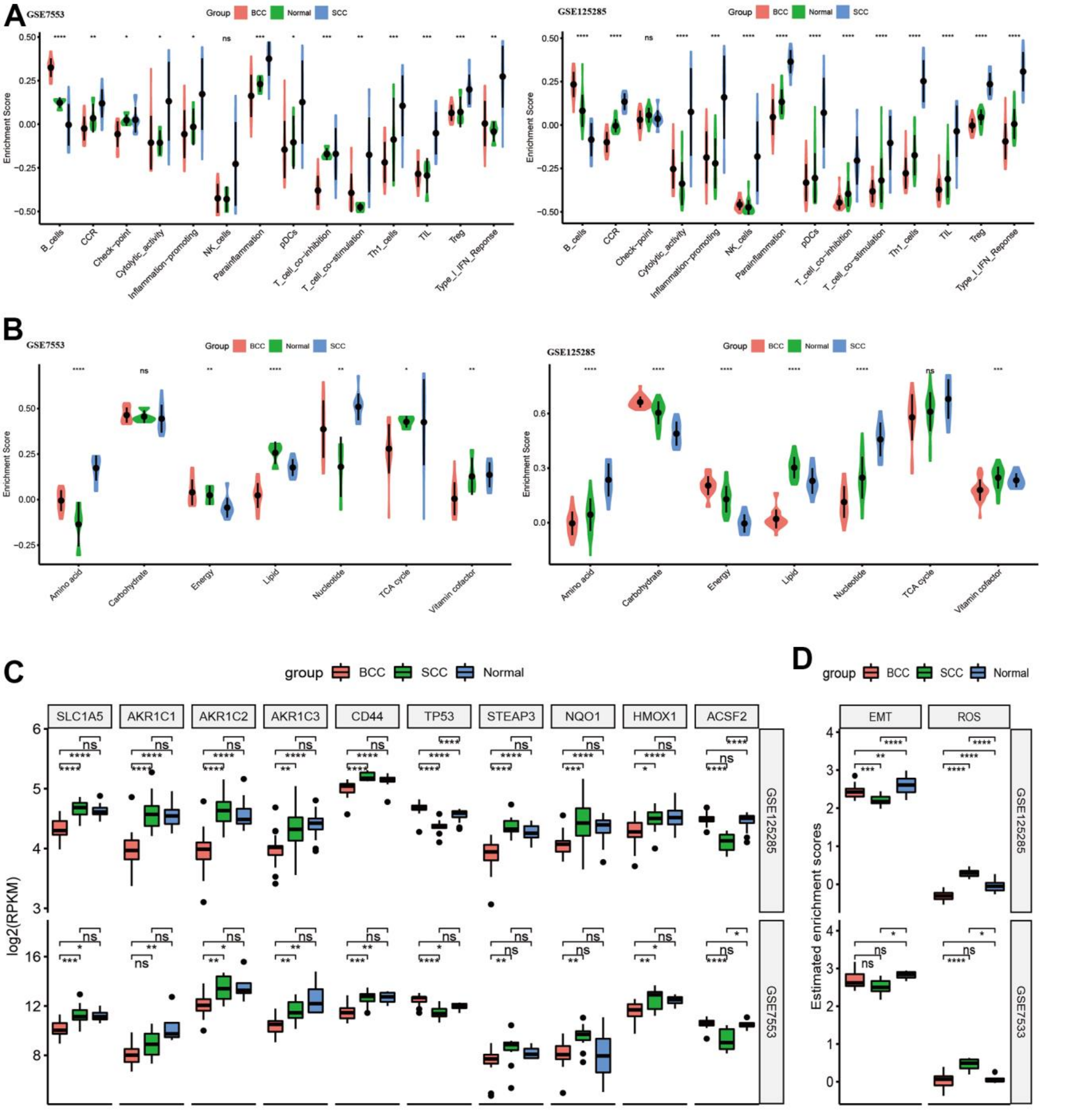

Supplementary Figure 4. The difference in GSVA(ssGSEA) enrichment results and ferroptosis genes expression of BCC, SCC and adjacent/normal groups. (**A**, **B**) Difference in LICA and TME metabolic function between BCC, SCC and adjacent/normal groups. (**C**) Difference in ferroptosis genes expression between BCC, SCC and adjacent/normal groups. (**D**) Difference in EMT and ROS estimated enrichment score between BCC, SCC and adjacent/normal groups. (*P<0.05,**P<0.01, ***P<0.001, ****P<0.0001).
