# Supplementary material for: Ferroptosis-related local immune cytolytic activity in tumor microenvironment of basal cell and squamous cell carcinoma
Source: Aging (Albany NY). 2022 May 2;14(9):3956–72. doi: 10.18632/aging.204057 (PMC9134950; doi:10.18632/aging.204057)
Supplement: Supplementary File 1 [file aging-14-204057-s002.pdf]

SUPPLEMENTARY FILE

Supplementary File 1: Difference in genes expression in DEG geneset of three important KEGG pathways between BCC and SCC.

Pathways list:

Basal cell carcinoma (hsa05217)

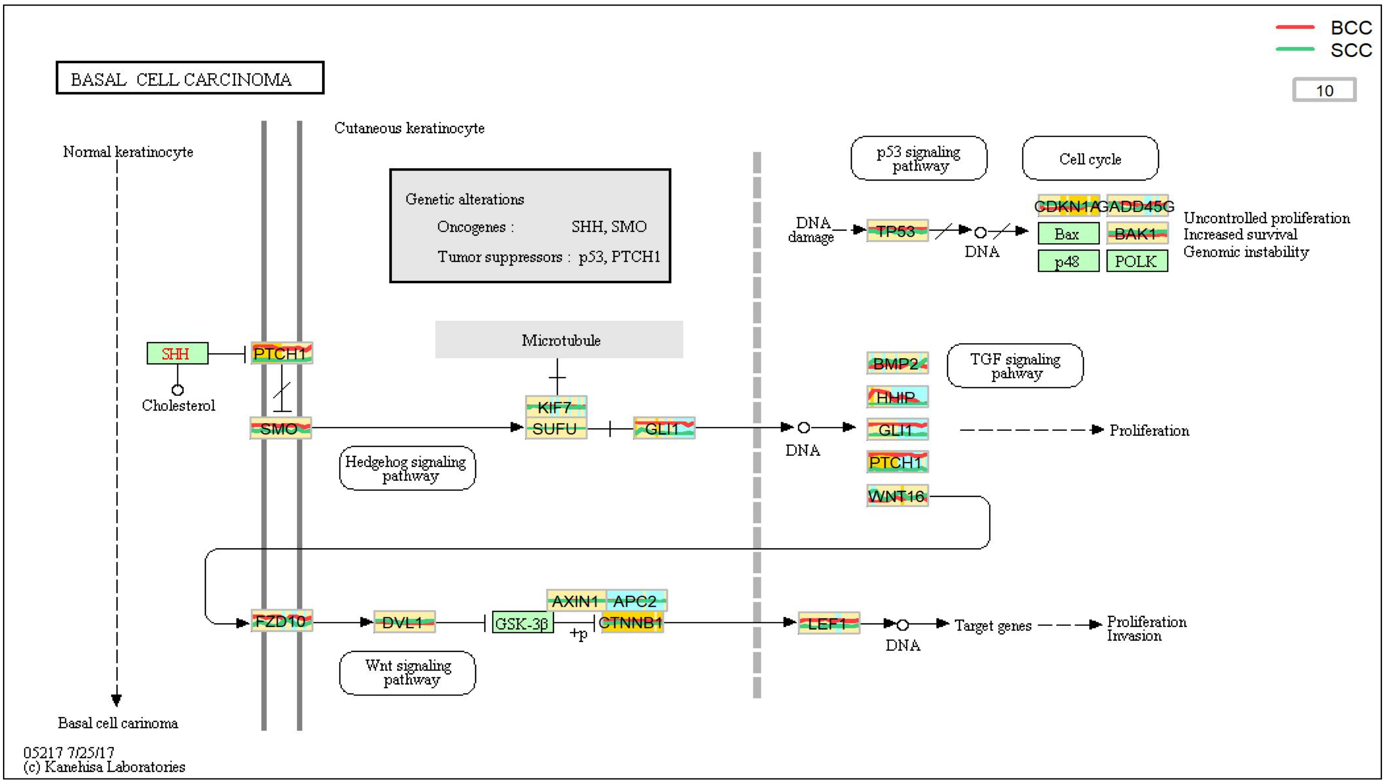

Ferroptosis (hsa04216)

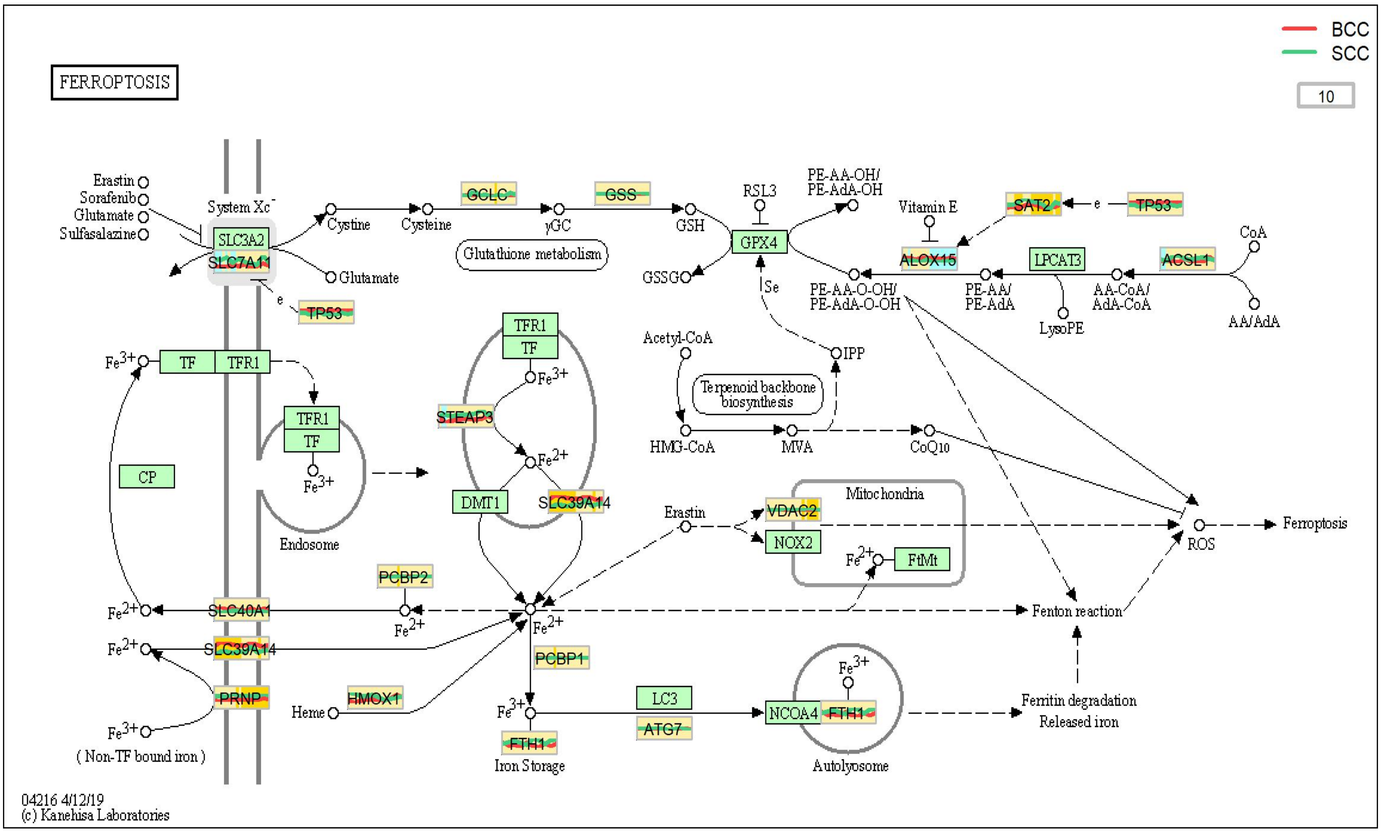

Endocytosis (hsa04144)

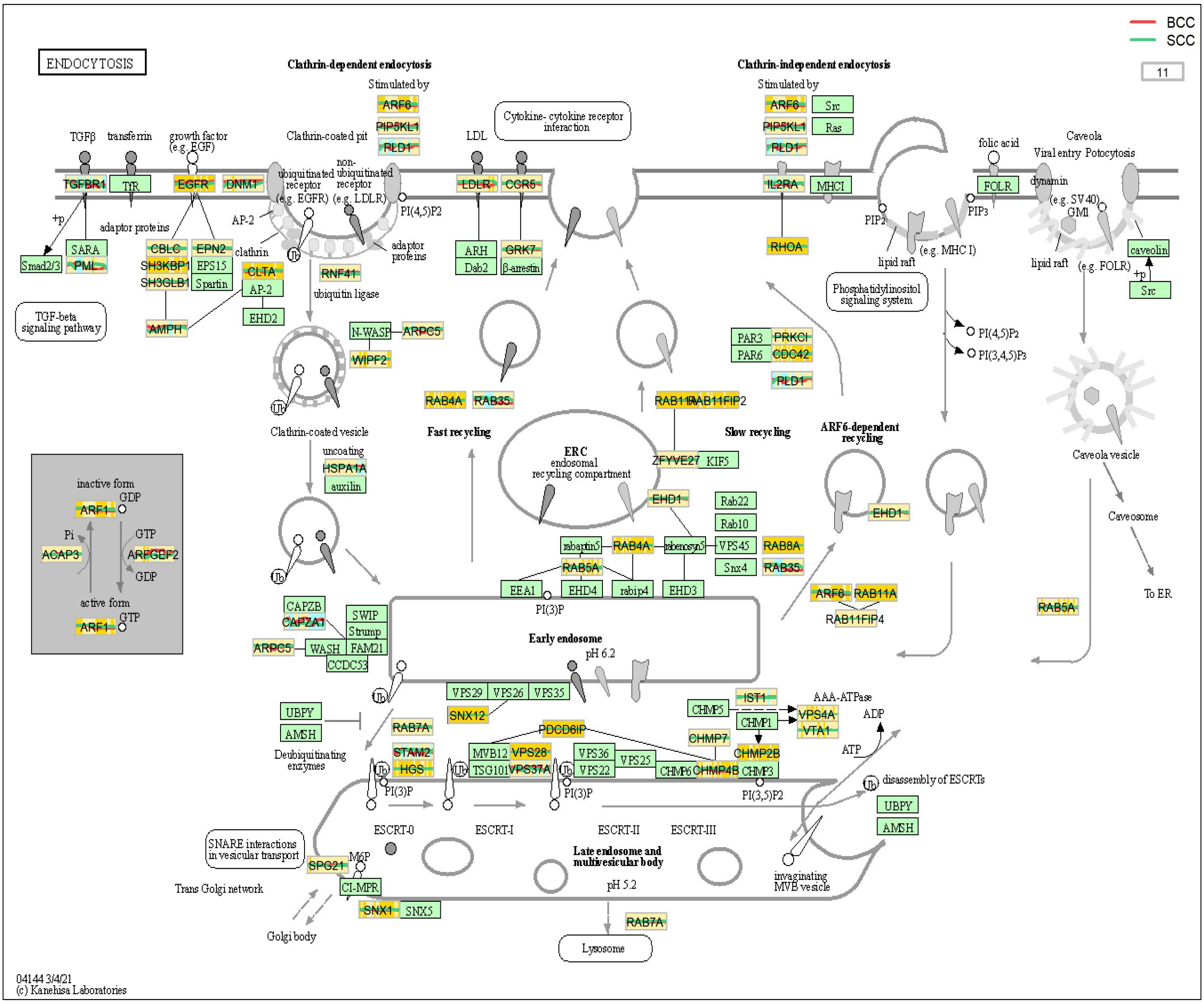

Notes: The genes marked with yellow or cyan background are the shared differentially expressed genes (DEGs) between BCC and SCC in the two datasets GSE7553 and GSE125285, and the yellow and cyan colors represent the proportion of samples with up-regulated and down-regulated expression of this gene, respectively. Genes marked with light green background are non-shared DEGs. The red or green curve represents the normalized value of the relative expression level of the gene in the BCC and SCC samples.
